# Prediction of Cognitive Load from Electroencephalography Signals Using Long Short-Term Memory Network

**DOI:** 10.3390/bioengineering10030361

**Published:** 2023-03-15

**Authors:** Gilsang Yoo, Hyeoncheol Kim, Sungdae Hong

**Affiliations:** 1 Creative Informatics and Computing Institute, Korea University, Seoul 02841, Republic of Korea; 2 College of Informatics, Korea University, Seoul 02841, Republic of Korea; 3 Division of Design, Seokyeong University, Seoul 02713, Republic of Korea

**Keywords:** electroencephalography, long short-term memory network, attention mechanism, cognitive load, deep learning

## Abstract

In recent years, the development of adaptive models to tailor instructional content to learners by measuring their cognitive load has become a topic of active research. Brain fog, also known as confusion, is a common cause of poor performance, and real-time detection of confusion is a challenging and important task for applications in online education and driver fatigue detection. In this study, we propose a deep learning method for cognitive load recognition based on electroencephalography (EEG) signals using a long short-term memory network (LSTM) with an attention mechanism. We obtained EEG signal data from a database of brainwave information and associated data on mental load. We evaluated the performance of the proposed LSTM technique in comparison with random forest, Adaptive Boosting (AdaBoost), support vector machine, eXtreme Gradient Boosting (XGBoost), and artificial neural network models. The experimental results demonstrated that the proposed approach had the highest accuracy of 87.1% compared to those of other algorithms, including random forest (64%), AdaBoost (64.31%), support vector machine (60.9%), XGBoost (67.3%), and artificial neural network models (71.4%). The results of this study support the development of a personalized adaptive learning system designed to measure and actively respond to learners’ cognitive load in real time using wireless portable EEG systems.

## 1. Introduction

The COVID-19 pandemic has caused significant disruptions to traditional classroom education worldwide, resulting in a surge in distance learning methods [1,2]. The rapid development of information technology (IT) has facilitated this transition by allowing students to continue their education from a distance. Consequently, traditional classroom education has gradually integrated online and distance learning methods, with distance learning emerging as a new trend in education [3,4]. Distance learning offers learners the flexibility to create a learning environment that transcends spatial and temporal constraints.

During the pandemic, many people were forced to work and study remotely, which has increased interest in developing methods for monitoring cognitive load levels in these settings. A recent issue related to cognitive load recognition has been its application to remote work and online learning. In this context, the challenge is the lack of face-to-face interactions, which makes it difficult to detect non-verbal cues that indicate cognitive load levels. Consequently, researchers have been exploring the use of physiological signals, such as EEG and eye tracking, to monitor cognitive load levels in real time.

Cognitive load is a measure of the mental effort required to complete a task and can be used to predict performance, fatigue, and stress levels. Cognitive load recognition is designed to improve human performance by identifying and monitoring cognitive load levels in real time. Therefore, cognitive load detection has numerous applications in various domains, such as healthcare, education, and aviation.

Driven by advances in computational neuroscience, research has been conducted to measure learners’ cognitive load based on the cognitive load theory. Cognitive load is one of the main causes of poor performance in a wide variety of tasks, including learning processes and associated thinking or reflection. If the degree of cognitive load of learners in learning or work processes is reflected, it can be used to develop adaptive instructional designs. However, most existing studies have focused on methods to estimate learners’ degree of cognitive load during the learning outcome stage [5,6]. A study investigating prefrontal cortex (PFC) hemodynamics using functional near-infrared spectroscopy (fNIRS) while performing n-back and random number generation (RNG) tasks with multiple cognitive loads suggested a relationship between subjective workload and brain activity [7]. In attempting to quantify the cognitive load, cognitive load modeling techniques using deep learning are also being studied, considering workload mechanisms and their impact on human performance [8].

Measuring the degree of cognitive load of learners after completing a learning experience has certain limitations. However, the measurement of these qualities during learning presents several challenges. To overcome these limitations, the development of adaptive models that provide instructional control to learners by measuring their cognitive load in real time has emerged as a promising approach. Real-time teaching feedback could facilitate active support that reflects changes in learning status and real-time applications to help develop adaptive instructional materials. These materials based on real-time measurement can manage learners’ cognitive load and participation in learning at an appropriate level during learning and help educators identify learners’ difficulties. To realize such real-time adaptive teaching, a method to measure learners’ cognitive load in real time during their learning experiences must be developed as a technical prerequisite.

In this study, we consider that learners’ cognitive load can be measured in real time using data on their physiological and psychological responses. Electroencephalography (EEG) is commonly used to collect these data. Hence, measuring cognitive load by collecting learners’ physiological data does not interfere with their learning experience. Cognitive load was measured using EEG analysis. EEG is the flow of electricity generated by signal transmission between brain nerves, and EEG analysis analyzes the frequency change in the EEG. Because EEGs exhibit different frequency wavelengths depending on mental activity, the degree of cognitive load can be measured by EEG analysis [9,10]. However, the generation of brainwaves is greatly affected by physical exercise and by differences in individual cognitive abilities. Noise in the signals may also pose some difficulties in interpreting information. Moreover, some authors have noted that EEG readings can be affected by other mental activities and that the continuous nature of the collected data poses notable difficulties in determining a person’s degree of cognitive load.

However, this approach can be used to develop a model to predict specific results using learner information by applying artificial intelligence-based methods such as machine learning. Friedman et al. explored various cognitive load prediction models based on machine learning using learners’ EEG measurement data. They compared and analyzed four machine learning algorithms (XGBoost, random forest, artificial neural network, and simple linear regression models) and reported that the XGBoost algorithm exhibited the highest predictive accuracy [11]. Machine learning algorithms may vary in prediction accuracy owing to variables such as the size of the training dataset. Similarly, the accuracy of artificial neural network algorithms varies with the number of hidden layers implemented in different models. Hence, a comparative analysis of the various algorithms is required. Therefore, we trained several machine learning models to predict cognitive load based on EEG data to compare their predictive performance.

In this study, we aimed to develop a model to measure learners’ cognitive load based on their neurophysiological reactions. Additionally, we are interested in creating personalized models that can account for individual differences in cognitive load responses. To this end, we developed a long short-term memory (LSTM)-based machine learning model to predict the degree of cognitive load using EEG data. To induce a measurable difference in cognitive load, we presented participants with video learning tasks of different difficulty levels and collected EEG data to compare the degree of understanding of the content that the participants showed during the tasks. Based on these data, we applied support vector machines, K-nearest neighbors, artificial neural networks, convolutional neural networks, deep belief networks, (recurrent neural network) RNN-LSTM, bidirectional LSTM, and bidirectional LSTM attention models to compare their performance in handling data most predictably and efficiently. A recent issue related to cognitive load recognition has been its application to remote work and online learning and the development of more accurate and personalized models to monitor cognitive load levels in these settings.

The remainder of this paper is organized as follows. Section 2 introduces previous studies related to cognitive load, and Section 3 explains the implementation of the proposed bidirectional LSTM combination model. Section 4 explains the analysis and results of the study, and Section 5 presents conclusions and future research directions.

## 2. Related Work

### 2.1. Cognitive Load

Methods of measuring mental workload include subjective methods using response forms filled out by participants, and objective methods, including the use of psychophysiological measurements [12,13]. One of the best ways to measure mental workload with a high temporal resolution is to utilize EEG data [11,12,13]. In this study, we propose an algorithm to explore the mental workload associated with multitasking activities using EEG measurements and to recognize different levels of mental workload.

According to the theory of cognitive load, learners’ management of cognitive resources is considered important for effective learning. Cognitive load theory argues that information processing that occurs in the learning process must be implemented within a limited capacity of working memory and that cognitive overload occurs if mental activity exceeds this limit [14]. The total cognitive load is composed of the sum of the extrinsic and intrinsic loads, of which the extrinsic load is considered to be lowered through efficient instructional design because it is a negative load owing to an incorrect design [15]. In contrast, because the intrinsic load is considered a positive load that helps form cognitive schemas, the total amount of cognitive load must be low for it to not be positive. For successful learning, appropriate teaching controls should be provided depending on learners’ individual characteristics to avoid imposing either an excessively high or low cognitive load for a given learning situation [16]. This argument of the cognitive load theory is related to the need for adaptive teaching. Teaching should be adjusted according to the level and characteristics of each learner. In particular, the expertise reversal effect of teaching guidance that does not meet the needs of learners can act as an unnecessary cognitive load, highlighting the need for adaptive teaching considering learners’ individual levels of knowledge [5]. Beginners can learn more effectively if they are provided with sufficient instructional guidance because they do not form mental schemas for certain learning topics. In contrast, for learners who have sufficiently developed a related schema, providing excessive instructional guidance hinders learning [17]. In other words, the application of adaptive teaching can optimize learners’ cognitive load to achieve more positive learning outcomes. Studies applying adaptive teaching have reported that groups presented with adaptive teaching methods showed significantly higher knowledge acquisition, shorter learning time, and higher teaching efficiency compared to groups that did not.

Various methods have been proposed to measure cognitive load; however, there is no absolute method. Brünken, Plass, and Leutner divided cognitive load measurement methods into two categories: distinctions between subjective and objective methods and those between direct and indirect methods [18]. In the subjective-direct method, the level of stress perceived by learners and the degree of task difficulty were measured using a questionnaire. In contrast, the subjective-indirect method adopts a self-reporting approach to evaluate the degree of mental effort that learners experience through a written questionnaire. Objective-direct methods include electroencephalogram measurements or double-task response time measurements, whereas objective-indirect methods include physiological characteristics or behavioral measurements. According to Brünken et al.’s classification, EEG measurements consider cognitive load directly, whereas physiological signals (sweat, pupil, etc.) indirectly measure cognitive load. In general, subjective questionnaires, double-task reaction time measurements, and physiological signal measurement methods have been used [19]. A representative subjective method involves asking the participants to respond to subjective questionnaires. This method involves self-reporting the difficulty of the task by learners based on their subjective experience with the mental effort they put in through a questionnaire. This approach is the most commonly used and provides a relatively simple measurement of the degree of cognitive load without requiring special equipment. However, it has a disadvantage in that changes in the degree of cognitive load that occur during learning cannot be observed, and it relies on subjective reports provided after the end of learning. In addition, previous studies have not reached a clear consensus on which aspects should be measured by subjective perception, such as task difficulty. Similarly, care should be taken when interpreting results according to various learning contexts. For example, in the case of task difficulty, Kalyuga and Sweller measured the total cognitive load, while DeLeeuw and Mayer (2008) argued that it was related to the essential load [5,14,17,20]. Objective methods include the double-task response time and physiological signal measurement methods. First, the dual-task response time method measures the cognitive load based on the speed at which learners respond to additional tasks presented while performing a given task [21]. In general, if the response rate to an additional task is high, the level of cognitive load involved in processing the initial task is low. The physiological signal measurement method checks the cognitive load by measuring learners’ physiological responses [22,23]. Because the physiological signal measurement method is based on objective data, it can be used to collect information in a relatively accurate and real-time manner without affecting task performance [24].

State-of-the-art works in cognitive load recognition involve using various physiological signals such as EEG, fNIRS, and ECG in developing models for predicting cognitive load. Researchers have used machine learning and deep learning algorithms to process these signals and classify cognitive load levels. However, despite the significant progress made in this field, challenges remain, such as high individual variability, noise, and poor generalization of models. Therefore, it is necessary to develop more accurate and robust models for cognitive load recognition.

The proposed method for cognitive load detection uses deep learning techniques and is motivated by the need for a more accurate and robust model that can address some of the challenges encountered by existing methods. Random forest, AdaBoost, SVM, XGBoost, and ANN are all traditional machine learning models that operate on fixed-length feature vectors. These models are often trained using labeled data and can predict unseen examples based on the patterns learned during training. Bi-LSTM, on the other hand, is a type of deep learning model that operates on sequential data, such as text or speech. Bi-LSTM is a variant of the long short-term memory (LSTM) network and is a type of recurrent neural network (RNN). Bi-LSTM has been successful in many natural language processing tasks, such as sentiment analysis, machine translation, and speech recognition. Unlike traditional machine learning models, Bi-LSTM can learn from the temporal relationships between inputs, which makes it well suited for tasks that involve sequential data. Bi-LSTM attention models can handle variable-length inputs and automatically extract relevant features from the input sequence, enabling them to capture complex patterns in the data.

Accordingly, the proposed method has the potential to improve the accuracy of cognitive load detection and can be applied to various real-world scenarios.

### 2.2. Preprocessing and Feature Extraction

Neural oscillations or brainwaves are electrical reactions that occur in the interaction between brain nerves and human mental activity, and these oscillations serve as indicators that reflect brain activity. EEG analysis considers changes in the intensity of electrical signals generated in the brain by frequency and is often used as a physiological signal measurement method to measure cognitive load. EEG measurements were made using the potential differences between the electrodes attached to the head. Electrodes can be attached to specific locations on the head to measure the EEG data in specific brain regions. EEG analysis generally analyzes the frequency of collected EEG signals by applying a Fast Fourier Transform (FFT). Brainwaves are divided into delta (0–4 Hz), theta (4–8 Hz), alpha (8–12 Hz), low beta (12–16 Hz), high beta (16–25 Hz), and gamma (25–50 Hz) waves, depending on their frequency. The intensity of brainwaves varies according to the state of human mental activity, and the degree of human cognitive load can be estimated based on this measurement. For example, alpha waves appear mainly in relaxation, beta waves appear in problem solving, and gamma waves appear mainly in more complex mental functions [25]. Considering these characteristics, we measured the constituent factors of cognitive load separately in terms of the degree of activation of the brainwaves by frequency.

The institutional review board (IRB) protects the rights and well-being of the subjects in life-oriented research. The proposed study, first, does not involve invasive behavior, such as drug administration and blood collection. Second, data were collected using only simple contact-measuring equipment that did not follow physical changes. Therefore, it corresponds to the IRB review that is not required in accordance with the regulations of the National Bioethics Policy Institute of Korea. The non-copyright dataset used in the experiment was obtained from Kaggle [26]. EEG signals were collected from 10 college students while they watched video footage. A total of 20 videos were provided, including 10 with and 10 without a mental load. Students wore single-channel wireless headsets (MindSet) to obtain EEG signals, which were measured on a 7-point scale from 1 to 7. The MindSet device measured the voltage between a forehead electrode and two electrodes (one for the ground and the other for reference) in contact with the ear. It provides an output of 0 for mental states and 1 for nonmental states. While the students watched a two-minute video, the EEG device emitted various previously listed signals. If the student was not ready at the beginning of the video, we removed the first and last 30 s of the video and analyzed only the middle 60 s of the EEG signal. The average of each firing interval was calculated to characterize the overall values. Several features were calculated to characterize the time profile of the EEG signal. Some of these distributions are typically used to measure the shape (minimum, maximum, variance, skewness, and kurtosis) of statistical distributions rather than time series. However, the small number of data samples (100 data points for 10 subjects who watched 10 videos each), including the aforementioned features, can overfit the training data and degrade the performance of the classification models. Accordingly, we used only the mean as a feature of the classifier. 

Table 1 shows the structure of the EEG dataset used for deep learning, including the number of samples, the number of channels, and the range of values for the maximum and minimum amplitudes. We preprocessed 11,388 data points and partitioned them into separate training and validation sets. Specifically, we allocated 75% (8541 data points) for training and 25% (2847 data points) for validation. 

### 2.3. LSTM-Based Recurrent Neural Network

**Figure 1:**
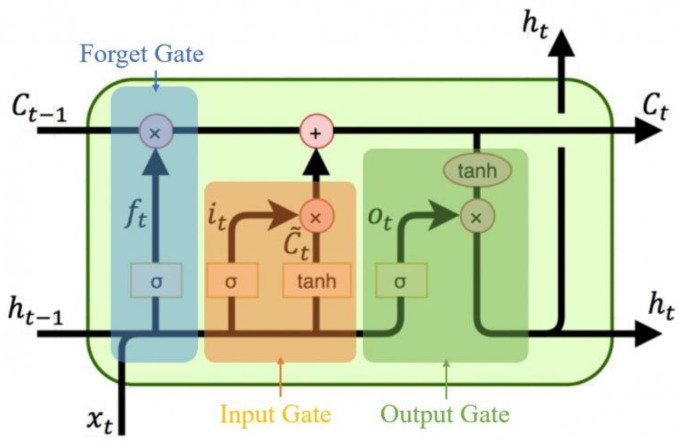
LSTM architecture comprises four components.

In contrast to CNN models, LSTM architectures are incapable of large-scale parallel processing. Unlike RNNs, they include input, output, and forget gates that can control the flow of data in the network at any time. The gates of the LSTM architecture can place memory blocks on hidden nodes to solve the long-term dependency problem of CNN models, although the memory block cannot remember all data. Moreover, when LSTMs are used in the pooling layer of a CNN, spatial and temporal features can be considered simultaneously, owing to the end-to-end structure. The LSTM layer compensates for the long-term dependence problem of the CNN. LSTMs are used to recognize the characteristics of sequential data and store them in memory using a variable called the cell state. As shown in Figure 1, the LSTM architecture includes input, output, and forget gates, which enable it to be variably controlled according to the characteristics of the input data.

The LSTM architecture consists of four components: input gate, forget gate, cell state, and output gate. The purpose of the input gate is to obtain new information using two features referred to as Rt and dt. Rt combines the previous hidden vector *h_t_*_−1_ with the new information *x_t_*. In other words, we multiply [*h_t_*_−1_, *x_t_*] by the new matrix *W_r_* and add the noise vector br. Then, we perform the same procedure for dt. Rt and dt multiply elements by element and import them into cell state ct. The slope of the forget gate is similar to that of the input gate; this component controls the limits of the values retained in memory. The cell state calculates the element multiplication between the previous cell states *C_t_*_−1_ and the forget ft. Then, we add the input gate rt multiplied by dt. The output gate is a symbol representing the output gate at *t*, and *W*_0_ and *b*_0_ are the weight and bias of the output gate, respectively. The hidden layer *h_t_* is moved to the next point or output *y_t_*.

### 2.4. Bi-LSTM

Bi-LSTM represents bidirectional long short-term memory. Bi-LSTM is a type of recurrent neural network (RNN) that is widely used for modeling sequential data. Unlike traditional RNNs that process input data in only one direction, Bi-LSTM models can process input data in both forward and backward directions simultaneously [27]. This makes them particularly useful for tasks such as natural language processing, speech recognition, and handwriting recognition, in which the context of each input data point depends on both past and future data points. The architecture of a Bi-LSTM model consists of two long short-term memory (LSTM) layers: one that processes input data in the forward direction and one that processes input data in the backward direction. Each LSTM layer has a series of memory cells that can store information over time and a series of gates that control the flow of information into and out of the memory cells. The gates are composed of sigmoid and tanh activation functions that determine the amount of information to be retained or discarded based on the relevance of the input data. By processing data in both directions, Bi-LSTM networks can capture both past and future contexts of a sequence, allowing them to better model complex dependencies and relationships within the data.

During training, the Bi-LSTM model was fed with the input sequences, and the weights of the network were updated using a backpropagation algorithm. The final output of the model was generated by concatenating the outputs of both LSTM layers, allowing the model to capture both the past and future contexts of the input sequence. 

Overall, the Bi-LSTM model showed promising results in various applications, demonstrating its effectiveness in capturing long-term dependencies and improving the performance of sequential data-processing tasks.

## 3. Materials and Methods

### Deep Learning-Based Cognitive Load Analysis Model

The model proposed in this study comprises a bidirectional LSTM and an attention mechanism to extract the positive and negative characteristics of the mental load. Contrary to conventional machine learning techniques, LSTM models are not capable of large-scale parallel processing, unlike CNNs. Instead, they utilize input, output, and forget gates to process the data. The gates have the advantage of being able to place a memory block on a hidden node. This can solve the long-term dependency problem of CNN models, although the memory block cannot remember all data. Moreover, when LSTM is used in the pooling layer of a CNN, spatial and temporal features can be considered simultaneously owing to its end-to-end structure. In addition, LSTM models can exhibit improved accuracy because they can equally model sequence vectors when predicting words. LSTMs provide sequential data characteristics and store them in memory using a variable called the cell state. This specialized architecture enables the data to be processed differently according to different situations by controlling the calculation process. Next, a single value is outputted using the sigmoid function in a fully connected layer called the dense layer.

A typical BCI system utilizes data preprocessing processes to remove noise, extract features, and classify the data to reflect characteristics and extract meaningful data from unprocessed brainwaves [28]. The classified information may be used as an instruction for device control or provided to the user. Because brainwaves are characterized by nonlinearity and high variability between individuals and situations, the implementation of stable and reliable BCI systems is challenging. In this study, we used an LSTM model to extract and classify cognitive load and related brainwave characteristics. The collected data were used as input to the LSTM model. The data to which the output value was assigned underwent a conversion process to make it suitable as input to the LSTM model.

**Figure 2:**
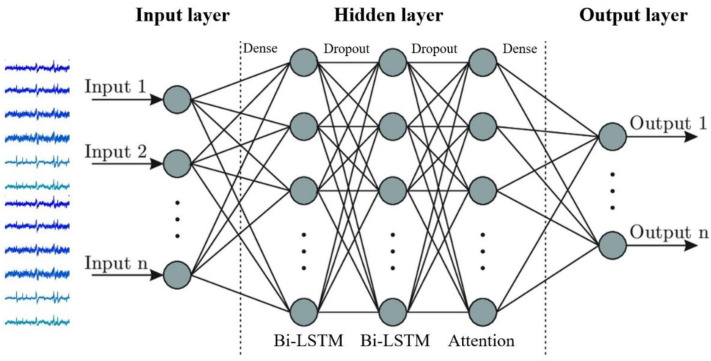
LSTM network structure with an attention mechanism.

In this study, we adopted a one-way LSTM layer followed by an attention mechanism to model the effect of the mental load generated at a given time on overall emotion. The attention mechanism is a learning method that weighs a part of the input that affects the output the most. Bidirectional LSTM layers are generally known to perform better when considering both the front and rear concealed states than unidirectional LSTM layers when using an attention mechanism. The overall structure of the proposed model is shown in Figure 2.

The first hidden layer included 128 neurons and used a bidirectional long short-term memory (LSTM) layer with a rectified linear unit activation function. Then, to avoid overfitting, we included a dropout with a probability of 0.2 and passed the data through a second bidirectional LSTM layer with 64 neurons and the ReLU activation function. After calculating the attention weight with the hidden state, which is the output of the second layer, the dimensionality of the data was reduced by passing through a layer with an output size of 16 and the first dense layer using the ReLU activation function. The final output is obtained by passing the data through the second dense layer, which has an output size of 1 and using the sigmoid activation function in the second classification and the softmax function in the third. In the second-stage classification, the output values are low or high for valence or arousal, and in the third-stage classification, the results are classified as low, middle, or high. The attention weights were processed in the following order: This method calculates the attention weight of the part of the input that affects the output; the higher the weight of the input part, the greater the value when the network is trained. The order of calculation is as follows: The hidden state vector calculated via the second bidirectional LSTM layer is multiplied by a randomly initialized attention weight, whose length is equal to the length of the hidden state vector. The output size of the second bidirectional LSTM layer was 64, with a total of 128, owing to the bidirectional architecture. Thus, the length of the attention weight is 128. The resulting value from this calculation was converted into a probability value through the softmax activation layer, and the transformed attention vector was combined with the first calculated hidden state vector to be calculated as the final attention output. Dense layer 1, which is connected to the attention layer, receives the corresponding attention output as input and reflects the part of the weight that is most important for future learning to produce more accurate results. The Adam optimizer was used, with a learning rate of 0.001. A cross-entropy loss function suitable for binary classification was also used. To measure accuracy, we adopted the Stratified *K*-fold cross-validation method with four iterations. Using this method, labels were distributed in a balanced manner for each fold; 75% of the data were used as the training set and 25% as the testing set for each iteration. Each iteration was trained for 30 epochs with an input batch size of 32. Each hyperparameter was optimized experimentally.

**Figure 3:**
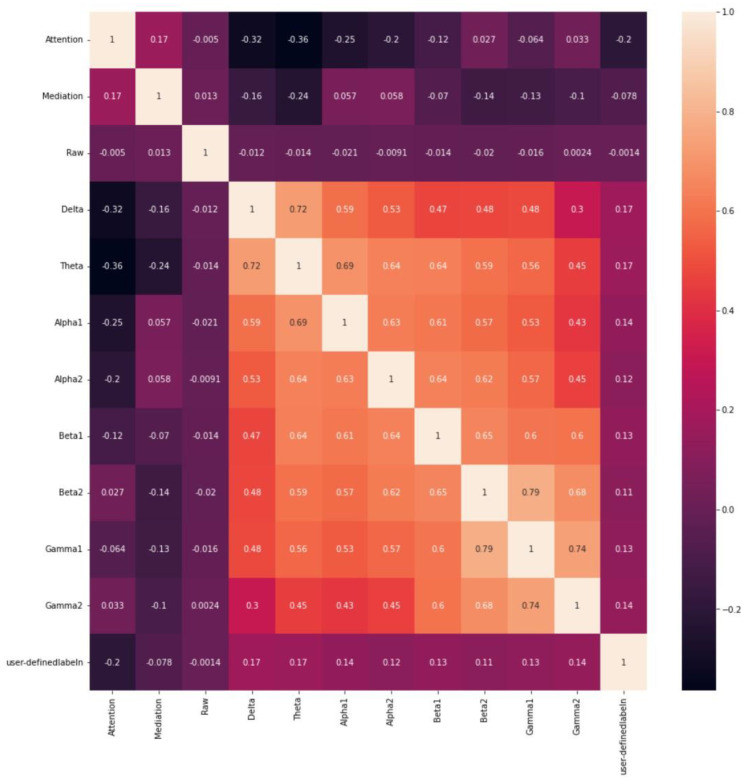
Results of the correlation analysis between variables.

Figure 3 shows the results of the analysis of the correlation between variables. In the heatmap, the *X*- and *Y*-axes were set to the same variable and plotted as points. Consequently, we observed a suitable correlation between Gamma1 and Beta2.

Table 2 summarizes the proposed LSTM model. The structure of the model was the same as that of the model implemented in Python. The ReLu activation function was used with a dropout of 0.2, fork of 100, and batch size of 10, and a sigmoid activation function was used in the last dense layer. 

## 4. Results

**Figure 4:**
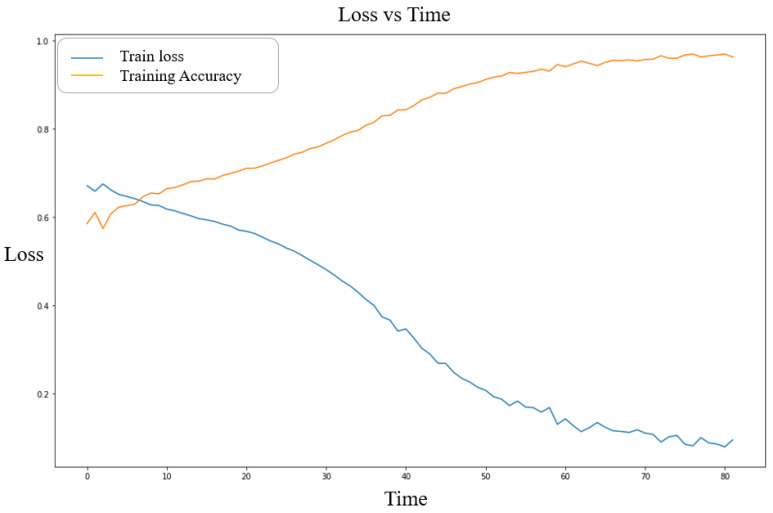
Loss and accuracy of the LSTM model.

The performance of the proposed Bi-LSTM model has a decisive influence on the overall system because control is performed based on accurate EEG feature extraction and classification according to the learning process. Loss and accuracy were used to verify the performance of the proposed LSTM model. The smaller the loss, the better because it indicates a difference from the result value. A categorical cross-entropy loss function was also used. Accuracy is the ratio of the total number of positive recognitions to that of negative recognitions. Thus, accuracy values closer to 1 indicate better performance. For the best training data, the proposed LSTM model exhibited a loss value of 0.08 and an accuracy of 96.9% over 76 epochs. For the verification data, the loss value was 0.3636, the accuracy was 84.8%, the loss value was 0.8289, and the accuracy was 87.11%. Figure 4 shows the loss and accuracy of the LSTM model according to data type. The *x*-axis represents the number of epochs, and the *y*-axis represents the accuracy according to the loss.

Table 3 presents the results of the comparison between the proposed method and the other algorithms. We evaluated the accuracy and F1-score of each model using both EEG training and test datasets. Our experimental results allowed us to identify the best model based on its accuracy and bias-variance balance. We can clearly identify the models with the highest scores. Specifically, the SVM model performed the worst, whereas the AdaBoost and random forest models performed similarly, with a performance 15.68% better than that of the most accurate ANN algorithm with an average accuracy of 0.871. The accuracy results of the models are as follows: two-way LSTM attention (0.871), ANN (0.714), RNN-LSTM (0.69), Bi-LSTM (0.6743), XGBoost (0.6733), AdaBoost (0.6431), and vector machine (0.6094). 

As shown in Table 4, we employed a technique to determine the optimal hyperparameters for all the models. By defining a grid of possible hyperparameter values and training the models with each combination of hyperparameters, the values that yielded the best performance in the validation set were identified. 

**Figure 5:**
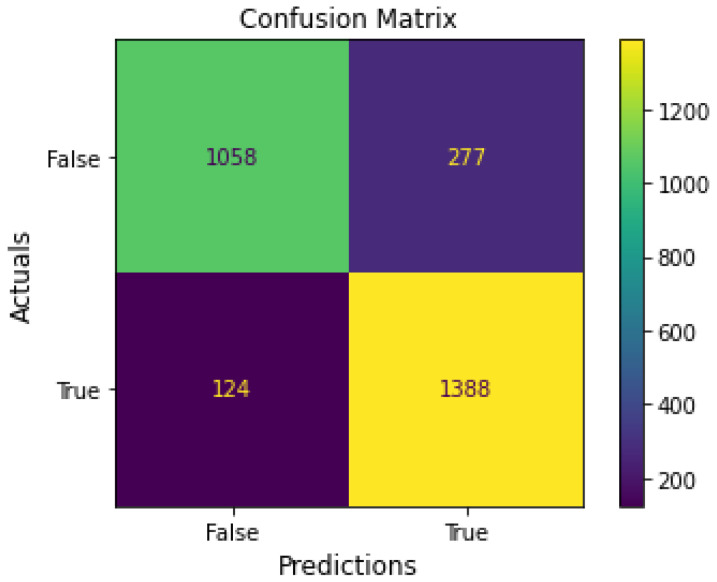
Confusion matrix of classification results.

A confusion matrix is a table for comparing predicted and actual values to measure prediction performance achieved through training [29,30]. As shown in Figure 5, the rows represent the correct answer class, and the column represents the predicted class. The confusion matrix, with 2847 data points, had a true negative, false positive, false negative, and true positive of 1058, 277, 124, and 1388, respectively.

Table 5 shows the precision, reproduction rate, and detailed classification report of the F1-score of the proposed model. Precision refers to the ratio of the number of samples belonging to the positive class among the samples shown to belong to the positive class, indicating a high precision of 0.918. Recall refers to the ratio of the number of samples detected to belong to the positive class among the samples in the actual positive class. The weight harmonic average of precision and recall is called the f-score, and the best result is false positive and false negative values close to 1. The classification report in Table 5 shows that 92% of the data predicted at 0 (mental load) were actually 0, and 83% of the data predicted at 1 (nonmental load) were actually 1. In addition, 80% of the actual cognitive load data was predicted to be cognitive load, and 93% of the non-cognitive load data was predicted to be non-cognitive load.

From the experimental results on algorithm comparisons, it was determined that traditional machine learning models, such as random forest, AdaBoost, SVM, XGBoost, and ANN, are best suited for tasks in which the input is a fixed-length feature vector. By contrast, Bi-LSTM is ideal for tasks that involve sequential data because it can learn from the temporal relationships between inputs, making it well suited for tasks such as EEG processing and cognitive load prediction.

The limitations of this study are as follows: First, the data used were insufficient to clearly reveal the difference in the degree of cognitive load in the composition of a given video based on participants’ understanding of the online learning video. We can consider a difference in the degree of cognitive load calculated by dividing the difficulty according to the understanding of the video; however, factors other than learning difficulty may have affected this value. Considering these limitations, the results of this study only suggest the possibility of determining the degree of cognitive load through machine learning using brainwave data. Accordingly, subsequent studies should clearly determine the differences in the difficulty of the learning tasks given to the experimental participants. Second, it is difficult to conclude that the accuracy of the model was represented well for all situations because the amount of student data collected was relatively small. Although the machine learning model was trained by integrating the data extracted from each experimental participant, the trained machine learning model would likely be unable to determine the degree of cognitive load universally because the number of participants was only 10. The accuracy of machine learning models depends on the amount of data available for training and verification. In particular, in the case of the ANN model used in this study, the accuracy was relatively high, and the possibility of overfitting was suspected. To confirm overfitting, data collected from additional participants were required. Third, the participants of the experiment were not evenly distributed. Accordingly, the ratio of the number of data samples used to train the artificial intelligence model is not equal. Because the training data are the basis for the model to determine the level of cognitive load, the uneven proportion of training data may have caused the accuracy of the model’s judgments to be inconsistent. Fourth, a real-time adaptive teaching model must be developed. In this study, we have presented EEG wavelength and electrode locations with a relatively large impact on EEG-based cognitive load determination and proposed an appropriate machine learning algorithm for the development of a cognitive load discrimination model. These research results only confirm the level of learners’ cognitive load, and it is difficult to confirm what support should be provided to learners from these data. To apply this to the educational field, an adaptive teaching model that provides appropriate teaching support according to learners’ cognitive load levels must be developed.

## 5. Discussion

The main reason for measuring the mental workload is to quantify the cognitive load that performs tasks to predict human performance. However, the existing method of evaluating mental workload presents a relationship between subjective workload and brain activity, making objective verification difficult. For instance, Agbangla et al. suggested a relationship between subjective workload and brain activity through PFC hemodynamics using fNIRS while performing n-back and RNG tasks with multiple cognitive loads, while Longo et al. suggested the possibility of mental workload modeling in EEG data using deep learning [7,8]. In this study, we trained an artificial intelligence model to determine learners’ levels of cognitive load using EEG data and confirmed the influence of different variables on cognitive load determination and the accuracy of the model with different machine learning algorithms. Applying bidirectional LSTM cyclic neural networks to classify student confusion regarding online course videos with EEG data showed that the bidirectional LSTM model achieved state-of-the-art performance compared to other machine learning approaches and showed suitable robustness as evaluated by cross-validation. As a result, gamma and alpha waves significantly influenced the determination of the discriminant model, and the bidirectional LSTM attention and ANN models exhibited the highest accuracy.

In this study, we propose a two-way LSTM recurrent neural network framework to detect a student’s mental load when watching online course videos. We implemented an attention-based LSTM deep learning model that effectively classifies cognitive load models by applying an attention mechanism, which is a state-of-the-art technology suitable for the mental load. The proposed model achieved an accuracy of 87.1% using EEG signals without a separate feature-extraction process. The results of a comparative analysis with other algorithms also showed that the accuracy of the proposed model outperformed that of other machine learning approaches, including a tomography LSTM model. The architecture of the bidirectional LSTM model helps leverage time-series capabilities for improved performance. An analysis of the contributions of each function to the model also confirmed that gamma and beta values are the most important for the cognitive load. In the future, the model should be trained with more EEG datasets, and the experimental results can be applied not only to learning but also to other EEG-related tasks, such as task evaluation and detection of drowsy driving.

In future studies, we intend to improve the accuracy of measuring cognitive load even in the lower class by applying a method to solve the data imbalance problem. In addition, for continuous cognitive load models, the degree of the mental load is important. Hence, we plan to apply a regression model to the last stage of the deep learning-based cognitive load model to analyze it in various ways.

## Figures and Tables

**Table 1:** EEG data set.

	Feature	Count	Max	Min
0	Attention	11,388	100.0	1.0
1	Mediation	11,388	100.0	1.0
2	Raw	11,388	1440.0	−2048.0
3	Delta	11,388	3,964,663.0	448.0
4	Theth	11,388	2,567,643.0	17.1
5	Alpha1	11,388	1,369,955.0	2.0
6	Alpha2	11,388	1,016,913.0	2.0
7	Beta1	11,388	840,994.0	3.0
8	Beta2	11,388	1,083,461.0	2.0
9	Gamma1	11,388	658,008.0	1.0
10	Gamma2	11,388	283,517.0	2.0
11	User-defined label	11,388	1.0	0.0
12	Age	11,388	31	24
13	Ethnicity	11,388	Han Chinese	Bengali
14	Sex	11,388	M	F

**Table 2:** The proposed LSTM model.

Layer Num	Type	Output Shape	Parameters
Layer 1	Input Layer	(None, 16, 1)	0
Layer 2	Dense	(None, 16, 64)	128
Layer 3	Dense	(None, 16, 128)	8320
Layer 4	Bidirectional LSTM	(None, 16, 512)	788,480
Layer 5	Dropout	(None, 16, 512)	0
Layer 6	Bidirectional LSTM	(None, 16, 512)	1,574,912
Layer 7	Dropout	(None, 16, 512)	0
Layer 8	Attention	(None, 16, 512)	528
Layer 9	Dense	(None, 16, 128)	65,664
Layer 10	Dense	(None, 16, 1)	129

**Table 3:** Classification result of comparison of the proposed method with other algorithms.

Classification Methods	Average Accuracy	F1-Score
Random Forest	0.6416	0.657
AdaBoost	0.6431	0.660
Support Vector Machine	0.6094	0.629
XGBoost	0.6733	0.686
ANN	0.7142	0.710
RNN-LSTM	0.6900	0.690
Bidirectional LSTM	0.6743	0.670
Bidirectional LSTM Attention	0.8710	0.870

**Table 4:** Grid search results for the best combination of parameters.

Models	Parameters (Grid Search)	Best Params
Random Forest	‘max_depth’: list (range (10, 20, 5)),	15
	‘n_estimators’: [50,100]	100
AdaBoost	‘algorithm’: [‘SAMME’,‘SAMME.R’]	‘SAMME.R’
	‘n_estimators’: [10,40,60,100,120,130,140]	120
SVC	‘kernel’: [‘rbf’]	‘rdf’
	‘C’: list (np.arange (0.5, 1.5, 0.1))	0.7
	‘gamma’: [‘scale’, ‘auto’]	‘scale’, ‘auto’
XGBoost	‘base_score’: list (np.arange (0.2, 0.5, 0.1))	0.4
	‘n_estimators’: [10,40,60,100,120,130,140]	60
	‘objective’: [‘binary:logistic’]	‘logistic’
ANN	Model hidden layer	{32, 16, 16}
	Dense (activation = ‘sigmoid’)	‘sigmoid’
	compile (loss = ‘binary_crossentropy’)	‘binary_crossentropy’
	optimizer = ‘adam’, metrics = [‘accuracy’])	‘adam’
	Dense (activation = ‘relu’,kernel_regularizer = ‘l2’)	‘relu’, 12

**Table 5:** Model performance of Bi-LSTM attention.

Bi-LSTM Attention	Precision	Recall	F1-Score	Support
0—Mental load	0.92	0.80	0.85	1360
1—Not mental load	0.83	0.93	0.88	1487
Accuracy	-	-	0.87	2847
Macro average	0.88	0.87	0.87	2847
Weighted average	0.87	0.87	0.87	2847

## Data Availability

The data presented in this study are available upon request from the corresponding author.
